# T Lymphocyte Maturation Profile in the EBUS-TBNA Lymph Node Depending on the DLCO Parameter in Patients with Pulmonary Sarcoidosis

**DOI:** 10.3390/cells10123404

**Published:** 2021-12-02

**Authors:** Elżbieta Rutkowska, Iwona Kwiecień, Joanna Bednarek, Rafał Sokołowski, Agata Raniszewska, Karina Jahnz-Różyk, Piotr Rzepecki

**Affiliations:** 1 Laboratory of Hematology and Flow Cytometry, Department of Internal Medicine and Hematology, Military Institute of Medicine, 04-141 Warsaw, Poland; ikwiecien@wim.mil.pl (I.K.); araniszewska@wim.mil.pl (A.R.); 2 Department of Internal Medicine, Pulmonology, Allergology and Clinical Immunology, Military Institute of Medicine, 04-141 Warsaw, Poland; jbednarek@wim.mil.pl (J.B.); rsokolowski@wim.mil.pl (R.S.); kjrozyk@wim.mil.pl (K.J.-R.); 3 Department of Internal Medicine and Hematology, Military Institute of Medicine, 04-141 Warsaw, Poland; przepecki@wim.mil.pl

**Keywords:** lymphocytes, effector T cells, central memory T cells, CD4+ cells, CD8+ cells, leukocytes, flow cytometry, EBUS-TBNA, DLCO

## Abstract

Sarcoidosis (SA) is a systemic granulomatous disorder of unknown etiology with lung and mediastinal lymph nodes (LNs) as the main location. T lymphocytes play important role in the formation of granulomas in SA, but still little is known about the role of maturation profile in the development of inflammatory changes. The aim of this study was to determine the CD4+ and CD8+ T cells maturation profile in LNs and in peripheral blood (PB) and its relation to disease severity expressed by diffusing capacity of the lung for carbon monoxide (DLCO). 29 patients with newly pulmonary SA were studied. Flow cytometry was used for cells evaluation in EBUS-TBNA samples. We observed lower median proportion of T lymphocytes, CD4+ T and CD8+ T cells in patients with DLCO< 80% than in patients with normal diffusion (DLCO > 80%). Patients with DLCO < 80% had lower median proportion of effector and higher median proportion of central memory CD4+ and CD8+ T cells than patients with DLCO > 80%. We reported for the first time that LNs CD4+ and CD8+ T cells maturation differs depending on the DLCO value in sarcoidosis. Lymphocytes profiles in LNs may reflect the immune status of patients with SA and can be analysed by flow cytometry of EBUS-TBNA samples.

## 1. Introduction

Sarcoidosis is a multisystem inflammatory granulomatous disease of unknown etiology most often manifested by enlarged lymph nodes (LNs) in the mediastinum and parenchymal changes in the lungs [1]. Inflammatory changes can also be found in the skin, LNs, eyes, liver nervous system and kidneys [2,3]. Multiorgan localization, varied picture and clinical course are the main causes of diagnostic difficulties [1]. Differentiation includes numerous systemic connective tissue inflammatory diseases, bacterial and viral infections [4]. The clinical picture must be obligatorily confirmed by histopathological examination showing the presence of granulomatous inflammation [5].

Accumulation of CD4+ T lymphocytes and macrophages transforming into epithelial cells and forming granulomas consists of epithelial and giant cells surrounded by lymphocytes and fibroblasts are characteristic features of sarcoidosis [6,7].

Lymphadenopathy is the most common symptom of sarcoidosis while lymphocytosis occurs in the bronchoalveolar lavage fluid (BALF) in most patients with lung sarcoidosis and exhibit an elevated CD4/CD8 ratio which has been associated with a diagnosis of sarcoidosis [8,9,10]. Several studies have suggested that the BALF CD4/CD8 ratio may complement the results of other tests when diagnosing sarcoidosis [11,12]. In contrast to BALF, the LNs in the mediastinum and hilars occupied by sarcoid granulomas have not been thoroughly tested by flow cytometry, despite the fact that the LNs are directly and widely involved in granulomatous inflammation [13]. In recent years, the mediastinal LNs are increasingly researched by use of ultrasound-guided transbronchial needle aspiration (EBUS-TBNA) to show granulomas in sarcoidosis [14,15,16]. EBUS is an adequate technique for pathological confirmation of clinically suspected sarcoidosis [17]. The EBUS-TBNA technique can provide high diagnostic performance in the diagnosis of sarcoidosis using flow cytometry analysis [15,18,19,20].

LNs act as a niche for various immune cells and the site of initiation of the immune response. Migrating recent thymic T lymphocytes (RTE) enter the LNs where they mature into naive T lymphocytes, which upon contact with an antigen, change into effector and central memory cells [21,22]. Effector cells directly are capable of inflammatory reaction, while central memory cells only after re-stimulating are activated, proliferate and form an immunological memory [23].

In our study diffusing capacity for carbon monoxide (DLCO) parameter was selected as the disease severity differentiating factor. The DLCO parameter is used to assess the function of the alveolar-capillary barrier that separates the air in the alveoli from the blood in the pulmonary capillaries and gives important information relating to the integrity and size of the alveolar blood membrane [24]. DLCO is widely used in the diagnosis of interstitial lung diseases such as sarcoidosis, allergic alveolitis, idiopathic pulmonary fibrosis and others such as congestive heart failure or anaemia [25,26,27]. A decline in DLCO value may indicate parenchymal involvement or a prognosis of pulmonary hypertension and may correlate with the severity and extent of SA [28,29,30].

The aim of this study was to determine the CD4+ and CD8+ T cells maturation profile in LNs and its relation to pulmonary SA severity depending on DLCO parameter.

## 2. Materials and Methods

### 2.1. Patients

The study included 29 patients with newly diagnosed pulmonary sarcoidosis. There were 14 women and 15 men; mean age: 45.0 ± 12.0 years; range (min–max): 24–68 years. Patients were in stages I–II of the disease: 12 patients in stage I and 17 patients in stage II. Each patient provided written informed consent (the Military Institute of Medicine Ethics Committee number 25/WIM/2018) before the diagnostic procedure including EBUS/TBNA and PB collection. Patients were hospitalized in Department of Internal Medicine, Pulmonology, Allergology and Clinical Immunology, Military Institute of Medicine (from January 2019 to July 2021). The characteristic of patients was summarized in Table 1.

A chest X-ray, computed tomography and functional tests were performed in all patients. EBUS-TBNA was made to evaluate cytologically the lesion and obtain material for analysing by flow cytometry. Primary sarcoidosis was confirmed by histopathology.

DLCO was measured for each patient according to American Thoracic Society/European Respiratory Society guidelines [31]. DLCO assesses the alveolar-capillary integrity and reflects the surface area and pulmonary capillary blood volume available for gas exchange [24]. DLCO value was the parameter that divided the group into: Group I consisting of 18 patients with DLCO > 80% and Group II consisting of 11 patients with DLCO < 80%.

### 2.2. Materials

During EBUS, suspected LNs were punctured starting from the most distal node station. All samples were assessed by an experienced pathologist to confirm the quality of the material obtained during the EBUS/TBNA procedure. The material was used for cytopathology staining and flow cytometry analysis. 4, 7 and 11 LNs groups were aspirated during the routine EBUS-TBNA procedure of sarcoidosis diagnosis. About 1 mL of LNs aspirates with 0.9% NaCl were collected in tubes containing K_2_EDTA and processed for flow cytometry method.

The routine test of white blood cells count (WBC) for all patients was performed using a haematological analyser Sysmex XN-1500 (Sysmex Corp., Kobe, Japan).

### 2.3. Flow Cytometry Analysis

Leukocyte and lymphocyte subpopulations were assessed by multiparameter flow cytometry with a panel of monoclonal antibodies using DxFLEX flow cytometry Beckman Coulter Company, Marseille Cedex 9, France). For the detection of lymphocyte T following antibodies were used: CD4-FITC (catalogue number: 345768, clone number: SK3), CD8-APC (catalogue number: 345775, clone number: SK1), CD3-PerCP-Cy5.5 (catalogue number: 332771, clone number: SK7). For the detection of NK cells following antibodies were used: CD16-APC-H7 (catalogue number: 560195, clone number: 3G8) and CD56-PE (catalogue number: 345810, clone number: MY31). For assessment of lymphocytes B CD19-PE-Cy7 (catalogue number: 341113, clone number: SJ25C1) was used. Additionally, HLA-DR-V450 (catalogue number: 655874, clone number: L243) and CD45-V500 (catalogue number: 655873, clone number: 2D1) antibodies were used (all mentioned above antibodies were from BD Bioscience).

**Figure 1:**
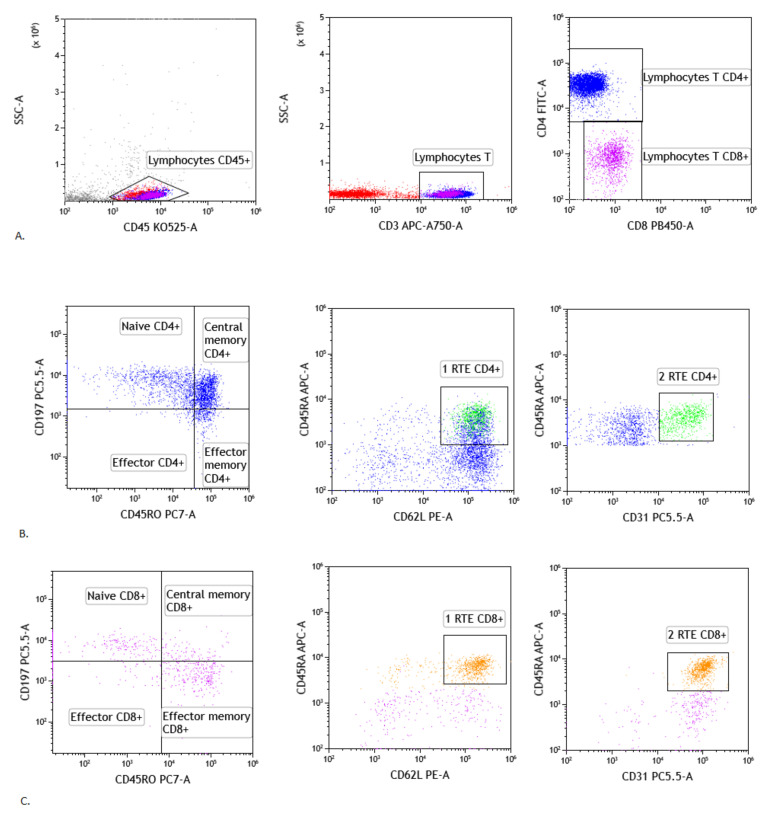
Gating strategy of T lymphocytes maturation in lymph node. (**A**) Selection of lymphocytes based on their SSC/CD45+ position, CD3 vs. SSC-A plot: Selection of lymphocytes T based on their SSC/CD3+ position. CD4 vs. CD8 plot: Selection of lymphocytes T CD4+ (blue) and CD8+ (purple) based on their CD4+ or CD8+ position. (**B**) Representative dot plots of T CD4+ subsets cells: naïve CD4+ T cells, effector CD4+ T cells, central memory CD4+ T cells, effector memory CD4+ T cells and recent thymic emigrants T CD4+ cells (gating strategy of RTE T CD4+ cells consist of two plots: 1RTE+ 2RTE - green), based on antibodies described in section: Material and Method. (**C**) Representative dot plots of T CD8+ subsets cells: naïve CD8+ T cells, effector CD8+ T cells, central memory CD8+ T cells, effector memory CD8+ T cells and recent thymic emigrants T CD8+ cells (gating strategy of RTE T CD8+ cells consist of two plots: 1RTE+ 2RTE - orange), based on antibodies described in Material and Methods).

For assessment the lymphocyte T CD4+ and CD8+ maturation were evaluated following populations: Recent thymic emigrants T cells: CD45RA+ CD62L+ CD31+ CD3+ CD45+ using antibodies: CD45RA-APC (catalogue number: 550855, clone number: -), CD62L-PE (catalogue number: 555544, clone number: -), CD31-PerCP-Cy5.5 (catalogue number: 566563, clone number: WM59), Naïve T cells: CD45RA+ CD197+ CD3+ CD45+ using antibodies: CD45RA-APC (catalogue number: 550855, clone number: -), CD197-PerCP-Cy5.5 (catalogue number: 353220, clone number: G043H7 BioLegend (San Diego, CA, USA), Effector T cells: CD45RA+ CD197- CD3+ CD45+ using antibodies: CD45RA-APC (catalogue number: 550855, clone number: -), CD196-PE (catalogue number: 551773, clone number: -), CD197-PerCP-Cy5.5 (catalogue number: 353220, clone number: G043H7 BioLegend (San Diego, CA, USA), Central memory T cells: CD45RO+ CD197+ CD3+ CD45+ using antibodies: CD45RO-PE-Cy7 (catalogue number: 560608, clone number: UCHL1), CD197-PerCP-Cy5.5 (catalogue number: 353220, clone number: G043H7 BioLegend (San Diego, CA, USA), Effector memory T cells: CD45RO+ CD197- CD3+ CD45+ using antibodies: CD45RO-PE-Cy7 (catalogue number: 560608, clone number: UCHL1), CD197-PerCP-Cy5.5 (catalogue number: 353220, clone number: G043H7 BioLegend (San Diego, CA, USA). We added to all samples: CD3 APC-A750 (catalogue number: 641415, clone number:SK7), CD4-FITC (catalogue number: 345768, clone number: SK3), CD8-V450 (catalogue number: 560347, clone number: RPA-T8) and CD45-V500 (catalogue number: 655873, clone number: 2D1). We assessed the lymphocytes maturation in the CD4+ and CD8+ cells [23]. Representative T lymphocytes maturation gating strategy in sarcoidosis patients were presented in Figure 1.

Samples were incubated for 20 min at room temperature. After two washing, cells were analysed within 2 h. For each sample, a minimum of 20,000 events were collected. Data were analysed with Kaluza C1.1 (Beckman Coulter Company, Marseille Cedex 9, France) and Infinicyt 1.8 Flow Cytometry Software (Cytognos, Salamanca, Spain).

### 2.4. Statistical Analysis

The Mann–Whitney test was used for comparisons between groups. For a statistical analysis was used The Statistica 12.0 software (TIBCO Software, Palo Alto, CA, USA) was used. The results are expressed as medians with interquartile range (Q1–Q3) and differences were considered statistically significant when *p* < 0.05. The Prism program (Version 5, GraphPad Software, La Jolla, CA, USA) was used to perform all analyses.

## 3. Results

### 3.1. Patients Characteristics

In all patients with suspicion of sarcoidosis with the presence of mediastinal lymphadenopathy, a chest X-ray and functional test were performed, showing the dilatation of the mediastinum. Computed tomography was performed showing altered LNs, densification of the pulmonary parenchyma, scar lesions of the pulmonary cavities or the presence of fluid in the pleural cavities. EBUS-TBNA was made for cytological evaluation of the lesion and obtaining material for evaluation by flow cytometry. Histopathological examination of the changed lymph node and the patient’s clinical appearance made the final diagnosis. There were 29 patients with newly diagnosed pulmonary sarcoidosis (clinical patient characteristic is presented in Table 1). Patients showed the most common symptoms of pulmonary sarcoidosis, such as: lymphadenopathy (100%), arthralgia (58.6%), fever (48.3%), cough (37.9%), dyspnea (13.8%) and pulmonary fibrosis (10.3%). Patients were divided according to the DLCO parameter (18 patients with normal DLCO parameter > 80% and 11 patients with DLCO < 80%). The patients characteristic dependent on DLCO parameter value was presented in Table 1. The group with DLCO < 80% had more clinical symptoms than the group with normal DLCO value. All patients in the study group were in I or II stage of sarcoidosis (stage I: 12, II: 17 patients). All patients did not have additional extrapulmonary forms of sarcoidosis.

We assessed leukocyte subpopulations and the maturation profile of T cells in the LNs nodes depending on the DLCO value.

Moreover, we presented the analyses for these cells’ subset in PB in Supplementary Materials. We did not observe differences in the main leukocytes’ subpopulation and T cells maturation subset in PB between patients with DLCO > 80% and DLCO < 80% (Supplementary Materials: Tables S1 and S2). Further analysis compared leukocyte subsets and maturation of T cells were made for LN samples.

### 3.2. Leukocyte Main Subsets in the Lymph Node

We analysed the main subpopulation leukocytes profile in LNs in patients with sarcoidosis depending on DLCO parameter. We observed a lower median proportion of lymphocytes T, both CD4+ and CD8+ cells in patients with DLCO < 80% than in patients with DLCO > 80% (Table 2).

### 3.3. T Cells Maturation Depending on the DLCO Parameter

When we analysed the maturation of CD4+ T cells, we observed lower median proportion of effector CD4+ T cells in patient with DLCO < 80% than in patients with DLCO > 80% (6.3 vs. 18.5%, *p* < 0.05). The median proportion of central memory CD4+ T cells was higher in patients with DLCO < 80% than in patient with DLCO > 80% (30.1 vs. 9.9%, *p* < 0.05) (Table 3).

**Figure 2:**
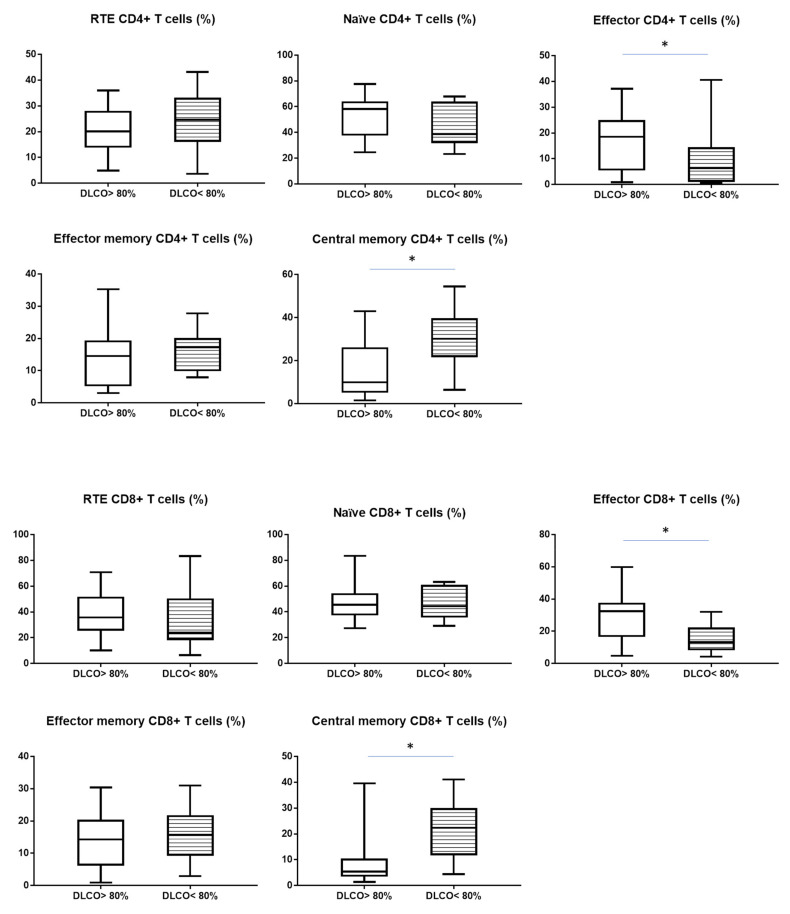
The differences in the median proportion of T CD4+ and CD8+ lymphocytes subsets: Recent thymic emigrants T cells (RTE), naïve T cells, effector T cells, central memory T cells and effector memory T cells between sarcoidosis patients with normal diffusion: DLCO > 80% (without stript) and with reduced diffusion: DLCO < 80% (with stript). Graphs show the median values and max-min (* *p* < 0.05 statistically significant.).

Analyzing the maturation of CD8+ T cells we observed a similar tendency as with CD4+ subsets. The median proportion of effector CD8+ T cells was lower than in patient with DLCO > 80% (13.2 vs. 32.4%, *p* < 0.05). The median proportion of central memory CD8+ T cells was higher in patient with DLCO < 80% than in patient with DLCO > 80% (22.4 vs. 5.4%, *p* < 0.05) (Table 3). All differences were shown in Figure 2.

## 4. Discussion

In this study, we focused on determining the cellular profile of the LNs in SA patients with an emphasis on the maturation of T cells. It is known that activated T lymphocytes are essential in the inflammatory process in SA and the production of inflammatory cytokines is crucial for the formation of the characteristic granuloma [6]. Most of the studies on the role of lymphocytes in the pathogenesis of sarcoidosis concern assessing the ratio of CD4 to CD8 T cells in BALF. Patients with pulmonary SA often have an increased number of lymphocytes with a high CD4/CD8 ratio (ratio of CD4+ T cells to CD8+ T cells) in BALF [10,12]. However, it has been shown that increased CD4/CD8 ratios in BALF are not sensitive and specific enough to replace histological confirmation of SA but only support the diagnosis [32]. The CD4/CD8 ratio is frequently used in the diagnosis of SA, however, this parameter does not reflect the severity of the disease [33] The specificity of pathogenic T cells in pulmonary SA are currently unknown and requires further research [34].

In contrast to BALF, T cells that infiltrate inflammatory SA LNs, have not been widely studied. Considering LN as a crucial site of the immune response, it seems important to evaluate the profile of LN cells to accurately reflect the cellular composition. Although the accumulation of CD4+ cells in the lung and other involved tissues is regarded as the distinctive immunologic feature of SA [35], there are no studies that show the distribution of the maturation profile of both CD4+ and CD8+ T cells, such as: RTE, naïve, effector, central memory and effector memory T cells in SA-affected LNs.

Therefore, it seemed interesting to thoroughly characterize the distribution of these cells, taking into account the degree of node involvement, patients with varying degrees of disease severity by DLCO values. In our previous research, we have shown that SA pulmonary patients with reduced diffusion (DLCO < 80%) had a higher proportion of fibrocytes and epithelial progenitor cells (EPC) than patients with DLCO > 80%. We indicated the participation of these cells in the pathogenesis of SA and their possible use as markers to assess the severity of the pulmonary SA [36].

In this study, we analysed the main subpopulation leukocytes profile in LNs and PB in patients with sarcoidosis dependent on DLCO parameter. We observed differences in T cells subpopulation between patient with DLCO > 80% and DLCO < 80% only for LNs (Table 2 for LNs and Table S1 for PB). We did not observe differences in CD4/CD8 ratio between study groups. We hypothesize that the widely used CD4/CD8 ratio parameter in SA did not distinguish patients with reduced pulmonary diffusion in both LNs and PB. Interestingly, our results showed that in LNs there was a decrease in the percentage of both CD4 and CD8 lymphocytes in patients with DLCO < 80%, possibly due to migration to the site of inflammation [37].

Akao K. et al. [16] have shown the CD4/CD8 ratio was significantly more elevated in LNs than in BALF. Moreover, they have presented that the CD4/CD8 ratio was significantly higher in stage I than in stage II in SA LNs. The regulatory T cells (CD4+, CD25+, FoxP3 high) proportion did not differ between lung cancer samples and sarcoidosis. Oda et al. [38] have reported that the CD4/CD8 ratio in SA LNs was lower than in the BAL fluid using immunohistochemical methods. In addition, Darlington et al. [39] have shown that the CD4/CD8 ratio in EBUS-TBNA samples from SA LNs was significantly lower than in BALF samples measured by flow cytometry.

In our study, we did not observe differences in CD4/CD8 ratio in SA LNs between groups of patients with different values of DLCO. The differences in LNs composition included changes only in the T subpopulations, while the other leukocytes did not differ between the studied groups (Table 2) Therefore, it seems interesting to investigate the variability in the maturation profile of T lymphocytes. T lymphocytes are one of the major components of granulation tissue formation. The SA granuloma consists of a core of epithelial cells and multinucleated giant cells (MGCs) surrounded especially by T cells as well as macrophages, dendritic cells and B lymphocytes [40]. Antigen-presenting cells transmit a signal to Th lymphocytes, activating them to secrete cytokines, which stimulates proliferation and recruitment of neutrophils and monocytes [41]. The maturation and activation of T cells can be viewed as an important step in granuloma formation. When we analysed the T maturation profile in LNs in patients with SA we noticed characteristic cellular composition in patients with diffusion-reduced lung capacity (DLCO < 80%).

Patients with DLCO < 80% had lower median proportion of effector CD4+ T and CD8+ T cells and higher proportion of central memory CD4+ T cells and CD8+ T cells than patients with DLCO > 80%. The presented profile of the T maturation in LNs patients with DLCO < 80% suggested a stronger reaction to the antigen than in a patient with normal diffusion lung capacity. The number of effector cells, both CD4 and CD8 decreased, with a marked increase in central memory cells ready for another contact with the antigen in LNs. The decrease in the number of effector cells is probably related to leaving LN and movement to the site of the inflammatory reaction. In previous studies, researchers have focused on only main lymphocyte subpopulations [42]. There are currently no studies examining the LNs composition of patients with SA. Lee N-S. et al. [43] analysed the maturation of T lymphocytes in the PB with SA. They have shown that patients with SA had increased numbers of central memory and effector memory CD4+ T cells with significantly decreased numbers naïve CD4+ T cells than the healthy group. In patients with SA, compared with healthy ones, it was found that CD4+ memory cells also had a significantly increased frequency of CD27 cells, which indicates chronic antigenic stimulation.

Interestingly, in our study, we noticed differences in CD8+ T cells maturation, with a tendency in cells composition such as CD4+ T cells maturation. The role of CD4 + T cells in the immunopathogenesis of sarcoidosis is well established, while less is known about cytolytic CD8+ T cells. In our study, similarly to CD4+ T lymphocytes, we noticed a decrease in the percentage of effector lymphocytes in LNs with an increase in CD8+ T central memory lymphocytes in patients with diffusion-reduced lung capacity (DLCO < 80%) than normal DLCO value.

We did not notice any differences for T cells maturation and main subpopulation of lymphocytes between groups with DLCO parameter <80% and >80% in PB. In contrast, Parasa R. V. et al. [44] demonstrated that cytolytic CD8+ T cells (CTL) in PB of sarcoidosis patients expressed relatively higher levels of effector molecules as well as increased CTL-dependent cytotoxicity, but compared SA to healthy controls. Sweiss N. et al. [45] have evaluated lymphocyte subsets in peripheral blood SA patients and observed significant lymphopenia involving CD4, CD8, and CD19 positive cells in comparison to healthy control. This finding may suggest that lymphocytes are depleted in PB due to infiltration to target organs. In another study in patients with SA in BALF was a significant relationship between the increase in the number of CD4+ T lymphocytes in BAL fluid and a reduction in the total number of CD4+ T cells in PB [46].

Our study provided the first report of main leukocytes and T cells maturation profile depending on DLCO parameter in sarcoidosis patients in LNs and PB. The above results indicate that the analysis of LNs SA will more accurately reflect the ongoing disease process than PB and provide a new direction for further research on SA pathogenesis.

## 5. Conclusions

We analyzed for the first time T cell subsets and T cells maturation profile in LNs SA with normal and reduced lung diffusion capacity. Decreased effector CD4+ and CD8+ T cells and increased central memory CD4+ and CD8+ cells in LNs were characteristic for sarcoidosis patients in the advanced disease phase. In summary, the severity of disease pattern in LNs SA based on the assessment of T cell maturation, combined with the accuracy and safety of EBUS-TBNA technique, make this approach very useful in the clinical evaluation of SA severity.

## Figures and Tables

**Table 1:** Characteristic of the study population with sarcoidosis.

	All Patients *n* = 29	DLCO > 80% *n* = 18	DLCO < 80% *n* = 11
Sex: F/M (*n*)	14/15	7/11	7/4
Age (mean ± SD years)	44 ± 11	44 ± 11	46 ± 12
DLCO (%) (mean ± SD)	82.8 ± 7.8	87.3 ± 6.0	75.5 ± 3.2
Clinical symptoms (*n*,%)			
- Cough	11, 37.9%	6, 33.3%	5, 45.4%
- Dyspnoea	4, 13.8%	1, 5.5%	3, 27.3%
- Fever	14, 48.3%	4, 22.2%	10, 90.9%
- Arthralgia	17, 58.6%	9, 50.0%	8, 72.7%
- Lymphadenopathy	29, 100.0%	18, 100.0%	11, 100.0%
- Pulmonary fibrosis	3, 10.3%	2, 11.1%	1, 9.1%
Stage of disease (*n*,%)			
I	12, 41.4%	8, 44.4%	4, 36.4%
II	17, 58.6%	10, 55.6%	7, 63.6%

Abbreviations: F, female; M, male; DLCO diffusing capacity for carbon monoxide.

**Table 2:** The differences in the median of white blood cells (WBC) count and median proportion of leukocytes subpopulation in lymph nodes (LNs) between sarcoidosis patients with normal diffusion: DLCO > 80% and with reduced diffusion: DLCO < 80%. Data expressed as median (Q1–Q3). A * marked *p* < 0.05 statistically significant.

[median (Q1–Q3)]	DLCO > 80% *n* = 18	DLCO < 80% *n* = 11	* *p* < 0.05 The Mann–Whitney U Test
WBC cells/µL	525 (163–1068)	546 (150–871)	*p* = 1.0000
Leukocytes subpopulation [%]			
Lymphocytes	90.6 (82.3–95.7)	90.8 (77.2–97.7)	*p* = 0.9120
T Lymphocytes	63.5 (57.6–69.0)	52.4 (42.2–59.9)	* *p* = 0.0144
CD4 cells	49.1 (45.9–51.5)	41.5 (31.2–45.9)	* *p* = 0.0243
CD8 cells	13.8 (10.7–16.3)	8.2 (7.3–12.6)	* *p* = 0.0494
Ratio CD4/CD8	3.3 (3.0–4.1)	3.6 (2.7–5.6)	*p* = 0.7070
B Lymphocytes	22.3 (17.1–33.7)	29.7 (25.0–44.9)	*p* = 0.9120
NK cells	2.3 (1.8–3.2)	1.4 (1.0–2.1)	*p* = 0.1115
Neutrophils	4.7 (0.8–12.2)	7.1 (1.8–19.3)	*p* = 0.4379
Monocytoid line cells	1.4 (0.8–2.3)	1.5 (1.0–2.9)	*p* = 0.5501

**Table 3:** Differences in the median proportion of T lymphocytes maturation in lymph nodes (LNs) between sarcoidosis patients with normal diffusion: DLCO > 80% and with reduced diffusion: DLCO < 80%. Data expressed as median (Q1–Q3). A * marked *p* < 0.05 statistically significant.

[median (Q1–Q3)]	DLCO > 80% *n* = 18	DLCO < 80% *n* = 11	* *p* < 0.05 The Mann–Whitney U Test
Maturation of CD4+ cells: [% of CD4+ cells]			
Recent thymic emigrants (RTE)	20.1 (14.6–27.2)	24.6 (16.4–32.8)	*p* = 0.3869
Naïve	58.1 (39.3–62.5)	38.6 (32.5–63.1)	*p =* 0.2962
Effector	18.5 (6.5–24.3)	6.3 (1.4–14.1)	* *p =* 0.0394
Effector memory	14.5 (5.6–17.7)	17.3 (10.1–19.8)	*p =* 0.2380
Central memory	9.9 (6.0–25.6)	30.1 (22.0–39.2)	* *p =* 0.0108
Maturation of CD8+ cells: [% of CD8+ cells]			
Recent thymic emigrants (RTE)	35.6 (27.7–48.9)	23.9 (18.8–49.7)	*p =* 0.6423
Naïve	45.4 (38.3–52.5)	44.4 (36.3–60.2)	*p =* 0.9120
Effector	32.4 (18.7–35.4)	13.2 (8.8–21.7)	* *p =* 0.0070
Effector memory	14.2 (6.7–19.8)	15.7 (9.5–21.5)	*p =* 0.3397
Central memory	5.4 (3.9–9.4)	22.4 (12.1–29.6)	* *p =* 0.0043

## Data Availability

The data presented in this study are available on request from the corresponding author. The data are not publicly available due to restrictions privacy and ethical.

## Supplementary Materials

The following are available online at https://www.mdpi.com/article/10.3390/cells10123404/s1, Table S1. The differences in the median of white blood cells (WBC) count and median proportion of leukocytes subpopulation in peripheral blood between sarcoidosis patients with normal diffusion: DLCO > 80% and with reduced diffusion: DLCO< 80%. Data expressed as median (Q1–Q3). A * marked *p* < 0.05. Table S2. The differences in the median proportion of T lymphocytes maturation (CD4+ and CD8+) subsets: recent thymic emigrants T cells (RTE), naïve T cells, effector T cells, central memory T cells and effector memory T cells in peripheral blood between sarcoidosis patients with normal diffusion: DLCO > 80% and with reduced diffusion: DLCO < 80%. Data expressed as median (Q1–Q3). A * marked *p* < 0.05.
