# Supplementary material for: T Lymphocyte Maturation Profile in the EBUS-TBNA Lymph Node Depending on the DLCO Parameter in Patients with Pulmonary Sarcoidosis
Source: Cells. 2021 Dec 2;10(12):3404. doi: 10.3390/cells10123404 (PMC8699538; doi:10.3390/cells10123404)
Supplement: Supplementary file 1 [file cells-10-03404-s001.zip › cells-1440016-supplementary.pdf]

Table S1. The differences in the median of white blood cells (WBC) count and median proportion of leukocytes subpopulation in peripheral blood between sarcoidosis patients with normal diffusion: DLCO> 80% and with reduced diffusion: DLCO< 80%. Data expressed as median (Q1–Q3). A * marked p< 0.05.

[median (Q1-Q3)]	DLCO> 80% n=18	DLCO< 80% n=11	* p<0,05 The Mann–Whitney U test
WBC cells/μL	5930 (5370-7200)	5440 (5180-6030)	p= 0.6354
Leukocytes subpopulation [%]			
Lymphocytes	24.9 (19.2-33.9)	23.0 (19.0-31.7)	p= 0.8749
T Lymphocytes	16.1 (12.5-25.5)	17.6 (13.1-27.5)	p= 0.9578
CD4 cells	10.1 (8.7-16.6)	12.1 (8.1-15.5)	p= 0.8749
CD8 cells	6.3 (3.7-8.9)	5.5 (4.8-11.9)	p= 0.9578
Ratio CD4/CD8	2.2 (1.3-2.5)	1.8 (1.3-2.1)	p= 0.4278
B Lymphocytes	2.8 (2.3-3.4)	2.2 (2.0-2.7)	p= 0.3131
NK cells	4.7 (3.7-5.5)	3.8 (2.2-4.8)	p= 0.4278
Neutrophils	66.3 (55.7-73.4)	65.3 (55.2-74.1)	p= 0.8748
Monocytes	7.1 (4.4-8.3)	8.6 (5.9-12.7)	p= 0.6354

Table S2. The differences in the median proportion of T lymphocytes maturation (CD4+ and CD8+) subsets: recent thymic emigrants T cells (RTE), naïve T cells, effector T cells, central memory T cells and effector memory T cells in peripheral blood between sarcoidosis patients with normal diffusion: DLCO> 80% and with reduced diffusion: DLCO< 80%. Data expressed as median (Q1–Q3). A * marked p< 0.05.

[median (Q1-Q3)]	DLCO> 80% n=18	DLCO< 80% n=11	* p<0,05 The Mann–Whitney U test
Maturation of CD4+ cells: [% of CD4+ cells]			
Recent thymic emigrants (RTE)	18.0 (14.9-27.6)	23.6 (21.0-28.7)	p= 0.3132
Naïve	41.0 (35.6-49.0)	46.1 (36.9-67.9)	p= 0.3676
Effector	1.9 (1.6-4.4)	1.7 (1.2-3.1)	p=0.5622
Effector memory	19.1 (10.4-27.1)	16.0 (8.7-23.7)	p=0.4923
Central memory	35.9 (28.4-44.7)	34.4 (22.0-38.4)	p= 0.3676

Maturation of CD8+ cells: [% of CD8+ cells]			
Recent thymic emigrants (RTE)	28.0 (18.3-49.7)	33.6 (23.6-51.1)	p=0.7925
Naïve	27.4 (18.1-49.0)	33.9 (25.4-62.2)	p=0.4278
Effector	29.5 (17.5-37.4)	27.2 (19.5-37.4)	p=0.958
Effector memory	25.2 (17.1-30.0)	28.5 (21.5-31.5)	p=0.8749
Central memory	8.1 (7.0-14.7)	9.4 (6.4-10.0)	p=0.9578
